# Plastic leachates impair picophytoplankton and dramatically reshape the marine microbiome

**DOI:** 10.1186/s40168-022-01369-x

**Published:** 2022-10-24

**Authors:** Amaranta Focardi, Lisa R. Moore, Jean-Baptiste Raina, Justin R. Seymour, Ian T. Paulsen, Sasha G. Tetu

**Affiliations:** 1 Climate Change Cluster (C3), University of Technology Sydney, Sydney, Australia; 2 School of Natural Sciences, Macquarie University, Sydney, Australia; 3 ARC Centre of Excellence in Synthetic Biology, Macquarie University, Sydney, Australia

**Keywords:** Plastic leachate, Microbial communities, *Synechococcus*, Picoeukaryotes, SAR11, Biogeochemical cycles

## Abstract

**Background:**

Each year, approximately 9.5 million metric tons of plastic waste enter the ocean with the potential to adversely impact all trophic levels. Until now, our understanding of the impact of plastic pollution on marine microorganisms has been largely restricted to the microbial assemblages that colonize plastic particles. However, plastic debris also leaches considerable amounts of chemical additives into the water, and this has the potential to impact key groups of planktonic marine microbes, not just those organisms attached to plastic surfaces.

**Results:**

To investigate this, we explored the population and genetic level responses of a marine microbial community following exposure to leachate from a common plastic (polyvinyl chloride) or zinc, a specific plastic additive. Both the full mix of substances leached from polyvinyl chloride (PVC) and zinc alone had profound impacts on the taxonomic and functional diversity of our natural planktonic community. Microbial primary producers, both prokaryotic and eukaryotic, which comprise the base of the marine food web, were strongly impaired by exposure to plastic leachates, showing significant declines in photosynthetic efficiency, diversity, and abundance. Key heterotrophic taxa, such as SAR11, which are the most abundant planktonic organisms in the ocean, also exhibited significant declines in relative abundance when exposed to higher levels of PVC leachate. In contrast, many copiotrophic bacteria, including members of the Alteromonadales, dramatically increased in relative abundance under both exposure treatments. Moreover, functional gene and genome analyses, derived from metagenomes, revealed that PVC leachate exposure selects for fast-adapting, motile organisms, along with enrichment in genes usually associated with pathogenicity and an increased capacity to metabolize organic compounds leached from PVC.

**Conclusions:**

This study shows that substances leached from plastics can restructure marine microbial communities with the potential for significant impacts on trophodynamics and biogeochemical cycling. These findings substantially expand our understanding of the ways by which plastic pollution impact life in our oceans, knowledge which is particularly important given that the burden of plastic pollution in the marine environment is predicted to continue to rise.

Video Abstract

**Supplementary Information:**

The online version contains supplementary material available at 10.1186/s40168-022-01369-x.

## Background

Marine plastic pollution is now recognized as a critical global issue that directly impacts life in our oceans via damage associated with ingestion, entanglement, and increased risk of disease [1–6]. It has been estimated that every year around 9.5 millions tons of plastic ends up as waste in the ocean [5], causing ~ US$13 billion annual damage to marine ecosystems [1]. Numerous factors including continued increases in annual plastic production rates, inadequate waste management systems, low recycling rates, and typically slow environmental degradation all indicate that this pollution will continue to impact the marine environment into the future [7–9].

The current research into the impact of marine plastic on microbiological processes has been focused on the microbial communities attached to plastic debris, termed the plastisphere [10]. These studies have provided insight into the abundance and diversity of microorganisms that colonize and form biofilms on different sizes and types of plastic particles and have revealed colonization by microbial assemblages that are distinct from the surrounding water [11, 12]. Many key planktonic marine microbes, including phototrophs (e.g., *Prochlorococcus*) and heterotrophs (e.g., Pelagibacter), which play major roles in the ocean carbon cycling [13, 14], tend to be scarce within the plastisphere [11] and are replaced by different heterotrophic and mixotrophic bacteria, such as members of the Pseudomonadales and Sphingomonadales orders. This indicates that plastic debris presents a new potential niche for a relatively small subset of marine microbes [15].

While the microbiology of the plastisphere is emerging, relatively little is known regarding the broader impact of marine plastic pollution, particularly the impact of chemicals known to leach from plastics into the surrounding environment. Laboratory experiments have suggested that dissolved organic carbon (DOC) can leach from plastics at a sufficient rate to stimulate the metabolism of specific heterotrophic bacteria [16]. However, in addition to DOC, plastic items have been shown to leach an array of organic and inorganic additives [17–19], such as UV stabilizers, plasticizers, metals, dyes, and flame retardants [20, 21] which may have inhibitory effects. More than 10,000 compounds leach from plastic, and of these, more than 2400 are considered substances of concern according to the European Union toxicity criteria [21]. Many of these additives have been detected in marine waters worldwide and have the potential to adversely affect marine life [20], including known carcinogens, mutagens, reproductive toxicants, and endocrine-disrupting chemicals [21]. Additives represent around 7% of the mass of non-fiber plastics [22], although their proportion can be considerably higher for some plastics, such as polyvinyl chloride [23]. Given that global quantities of ocean plastic are expected to reach ~ 250 million tons by 2025 [24], this would represent around 17 million tons of chemical additives. Thus, there is a clear need to assess how these leached substances affect marine organisms.

Evidence for plastic leachate toxicity has been demonstrated in laboratory cultures of marine eukaryotic algae [17, 19, 25] and photosynthetic bacteria [26, 27], but little is known about their effect on natural communities. Here, we performed a microcosm experiment to investigate the impact of polyvinyl chloride (PVC) plastic leachate on a marine microbial community from coastal shelf waters of eastern Australia (Fig. S1), classified as a productive temperate neritic environment [28]. We examined the impact of exposure to two concentrations of PVC leachate (1% and 10% dilutions) to test whether the toxicity observed in previous lab-based studies on the marine cyanobacterium *Prochlorococcus* [26, 27] is widespread among marine microbes, particularly the photosynthetic component. We also included two zinc chloride concentrations (0.13 mg/L and 1.3 mg/L) in the experimental setup since zinc is a common plastic additive and was identified as the most abundant inorganic component of PVC leachate in previous studies [17, 26]. Zinc has also been shown to adversely impact the growth and photosynthesis of specific marine microorganisms [29]. PVC is one of the most widely produced plastics, and the manufacture of PVC items involves a range of functional additives often present at high proportions, which may subsequently be released into the environment [23]. Therefore, PVC is an ideal plastic polymer to study the effects of leachates. Our findings demonstrate that exposure to PVC leachate and zinc has a significant negative impact on both prokaryotic and eukaryotic primary producers and stimulates the growth of a variety of heterotrophic microorganisms, resulting in communities with distinctly different functional capabilities.

## Methods

### Leachate preparation

Plastic leachate was prepared as per our previous study [26]. Briefly, commercial PVC black matting was purchased from a home goods shop (Daiso) and stored in its wrapper until leachate preparation. PVC was then cut into squares of ~ 1 cm^2^, weighed, then added at a concentration of 5 g per 100 mL into sterile Turks Island Salt artificial seawater media salt base [30] in acid-washed flasks, and left for 6 days at 22 °C on a shaking incubator (100 rpm) with constant illumination to allow leaching to occur. To remove all PVC plastic particles and possible bacterial contaminants from the plastic leachate stock, we filtered the contents of each flask through a Steritop bottle top filter (0.2-μm polyethersulfone (PES) (Millipore)) (Fig. S1). The stock was stored at 4 °C and used within 3 days of preparation. The stock was added aseptically to the experimental bottles at a final concentration of 1% and 10% of the initial concentration (i.e., 0.5 g/L and 5 g/L, respectively). The concentrations tested in this work were selected based on our previous experiment looking at the effects of PVC leachate on *Prochlorococcus* [26] and represent equivalent or lower leachate concentrations (both in terms of weight of material used to make the stock solution and the concentrations tested) to what has been used in other plastic leach toxicity studies [16, 17].

### Seawater collection and experiment setup

Surface seawater was collected from the Integrated Marine Observing System (IMOS) National Reference Station (NRS) located at Port Hacking, Australia (− 34.116S, 151.219E) on November 19, 2019. Water was collected in 20 L acid-washed containers from just below the surface (< 0.1 m) using an in situ pump, with additional sensor data (chlorophyll fluorescence, temperature, oxygen) obtained from an SBE 911 plus CTD deployed alongside. Samples for nutrient concentration were collected as part of the NRS monthly sampling effort, and the analyzed nutrient data was obtained from the Australian Ocean Data Network (https://portal.aodn.org.au).

Once back in the laboratory (~ 3 h from collection), water was prefiltered through a sterile 60-μm nylon mesh (Millipore) to remove larger grazers. Microcosm experiments were then established using 4 L acid-washed Nalgene polycarbonate bottles. Four separate treatments were set up, comprising two concentrations of particle-free PVC leachate and two concentrations of added sterile ZnCl_2_ (0.13 mg/L and 1.3 mg/L) alongside a control comprising the filtered seawater with no additions. PVC leachate exposure treatments used PVC leachate added to the final concentrations of 1% (PVC1) and 10% (PVC10), to correspond with previously used concentrations [26]. The effect of ZnCl_2_ exposure was investigated alongside PVC leachate, as this is a common plastic additive and was found to be highly enriched in plastic leachate in our past work [26, 27] as well as in other plastic leachate studies [17, 31, 32] and has been shown to adversely impact marine *Synechococcus* and *Prochlorococcus* in lab experiments [29]. The zinc (Zn) concentrations selected here were based on the Zn concentrations measured from previous PVC leachate batches using inductively coupled plasma mass spectrometry (ICP-MS) (Additional file 2: Table S5b). Due to batch variability of the PVC item used, however, Zn concentrations that leached from the PVC mat differed from amounts of zinc chloride used for low and high Zn experimental bottles (PVC 1% had 0.04 mg/L, roughly one-third of the ZnL 0.13 mg/L, while the 10% PVC treatment contained 0.36 mg/L, an intermediate level between the low and high Zn treatment).

Once microcosms were set up, all bottles were incubated in external tanks at the Sydney Institute of Marine Science (SIMS) with constant water recirculation from 4 m below the surface to mimic in situ ocean temperatures and using a screen mesh covering, which provided a 50% reduction in incident light to mimic below surface light conditions. The experiment was set up in quadruplicate for each PVC and Zn concentration as well as for the control, resulting in 20 polycarbonate bottles incubated in four tanks, with each tank containing a set of replicate experimental bottles, and incubated for 6 days (Fig. S1).

### Trace metal analysis composition

Trace metal analysis was performed on an ICP-MS at the Elements Analysis Facility, a National Association of Testing Authorities, Australia (NATA)-accredited facility, at Southern Cross University, NSW, Australia. Triplicate samples of the undiluted leachate and seawater were collected at the beginning of the experiment (day 0) for metal analysis. At the end of the experiment (day 6), three of the four replicate bottles for each condition were randomly chosen to sample for metal analysis. For each metal analysis sample, 20 mL of seawater or leachate was collected in a polypropylene 30-mL bottle and fixed with a final concentration of 1% high-purity nitric acid (Sigma). All plasticware used for trace metal work was acid-washed and rinsed thoroughly with ultrapure MilliQ water. Each polypropylene bottle was double rinsed with the sample prior to sample collection. At the elemental analysis facility, each sample was analyzed following the approved standard methods APHA 3125, which includes the analysis of reference samples of the trace metal of interest at the beginning and at the end of the run, plus running ultra-trace purity grade artificial seawater solution blanks before and after each set of triplicate samples to confirm blanks are consistently zero or below the instrument detection limit. This same protocol was performed on samples with added ZnCl_2_, but for these samples, only Zn was measured.

### Flow cytometry

Two 1-mL samples were taken daily from each bottle for flow cytometric analysis, with samples collected at approximately the same time (8.30 am) each day. One sample was analyzed immediately to check the stability of the photosynthetic community, and the second was fixed with a final concentration of 0.25% glutaraldehyde and stored at − 80 °C until further analysis. Samples were analyzed on a Cytoflex S flow cytometer (Beckman Coulter) using two different lasers (violet 405 nm and blue 488 nm). Photosynthetic communities were identified based on the specific intensity of chlorophyll (Chl) and phycoerythrin (PE) autofluorescence excited by the blue laser, forward light scatter (FSC), and side angle light scatter (SSC) from the blue laser. Each sample was run for 2 min at a stable flow rate of 30 μL/min. Flow cytometry analysis identified three different eukaryotic communities, photosynthetic picoeukaryotes (PEUK), photosynthetic nano-eukaryotes (NEUK) and phycoerythrin-rich eukaryotes (PE-EUK), and the cyanobacteria *Synechococcus* (Fig. S2).

To enumerate the heterotrophic bacteria and viruses, the samples were diluted 1/10 with 0.02-μm filtered (GE Healthcare) TE buffer and stained with 1X SYBR green I (Sybr) (Invitrogen) final concentration. For quantifying heterotrophic bacteria, the Sybr-stained samples were incubated in the dark for 10 min, then analyzed. For quantification of viruses, replicate Sybr-stained samples were incubated at 90 °C in the dark for 10 min, cooled for 5 min in the dark, then analyzed, as per published protocols [33]. All Sybr-stained samples were run for 2 min at a constant flow rate of 10 μL/min. Viral and bacterial populations were discriminated based on the intensity of the DNA stain (SYBR green fluorescence) against the Violet SSC (405 nm). Violet SSC was preferred to the blue SSC (488 nm) for better resolution for smaller particles (including different viral groups). To account for the possible presence of instrument noise, multiple blanks, prepared the same way as the samples, were run and recorded at the beginning of the analysis and after every eight samples. Counts in the blanks were always negligible compared to the counts in the samples and were subtracted for bacteria and virus counts. Consistent gating strategies were applied to all the samples, which allowed the discrimination of up to 5 different viral populations (Virus1, Virus2, Virus3, Virus4, and Virus5) and two heterotrophic bacteria population (HDNAb, LDNAb) (Fig. S3), consistent with previously published population identifications [34].

### Chlorophyll a fluorescence methodology

Samples for total chlorophyll fluorescence analysis were collected in triplicate at the beginning from the seawater used to start the experiment and at the end of the experiment from each experimental bottle; 250 mL of seawater was filtered on 25-mm GFF filters (Millipore) and stored at − 80 °C until extraction. Chlorophyll was extracted by placing filters in 4 mL of 90% acetone overnight at − 20 °C, sonicating for 7 min in ice water in the dark, then centrifuging at 5000 RPM at 4 °C for 5 min. The fluorescence emission at 664 nm of each supernatant, minus that at 750 nm, was measured using an Agilent Cary Eclipse Fluorescence Spectrophotometer. A chlorophyll standard curve was made from chlorophyll extracted from a marine *Synechococcus* culture in the same method as above and quantified on a Jasco V-730 Spectrophotometer following standard equations [35].

### Photophysiology measurements

To monitor the health of the photosynthetic community, fluorescence yields of fresh samples were run daily on a Soliense fast repetition rate fluorometer (FRRF). To deliver a single turnover saturation of the PSII, a flash sequence of 100 subsaturation flashlets with a 1.1-μs duration and 2.5-μs interval followed by a relaxation phase of 127 flashlets with a 1.6-μs duration and 20-μs interval was applied from a blue LED excitation source [36, 37]. The built-in curve fitting software on the Soliense was used to derive different fluorescence parameters from the recorded data, such as minimum fluorescence (*F*_*o*_), maximum fluorescence (*F*_*m*_), and maximum photochemical efficiency (*F*_*v*_/*F*_*m*_). Data was collected, and the mean of the blank measurement (obtained from the analysis of multiple 0.2-μm filtered seawater samples) was subtracted for all samples.

### DNA extraction

Samples for community and functional diversity analysis were collected at day 0 and at day 6 of the experiment. Three liters of seawater were filtered on a 0.2-μm polyethersulfone (PES) pore diameter filter (Millipore), and the filter was stored at − 80 °C until extraction. DNA was extracted using the DNeasy PowerWater kit (QIAGEN) following the manufacturer’s recommendations. DNA quality and yield were checked on a NanoDrop ND 3000 and on a Qubit Fluorometer 3.0, respectively.

### Amplicons sequencing and analysis

Amplification and sequencing of the 16S rRNA and 18S rRNA were used for the taxonomic analysis of bacterial and eukaryotic communities. Briefly, once extracted the DNA was amplified using 16S rRNA V1-V3 primers 27F-519R [38] for bacteria, and 18S rRNA V4 primers for eukaryotes, following the Australian microbiome initiative protocols (https://www.australianmicrobiome.com/protocols/), with both sets containing barcodes to enable multiplex sequencing on the Illumina MiSeq platform. Amplification procedures for the 16S and 18S rRNA also followed the Australian microbiome initiative protocols. Briefly, PCR reactions were set up in 96-well plates, and 16S rRNA and 18S rRNA amplicons were generated in 25-μL reactions using the hot start high fidelity Taq polymerase (Qiagen), following the manufacturer’s recommendation for reagent final concentration. Amplification conditions for the bacterial primers were 10 min at 95 °C, 35 cycles of denaturation 94 °C for 30 s, annealing 55 °C for 10 s and elongation 72 °C for 45 s, and a final extension step at 72 °C for 10 min.

For the 18S rRNA primers, several different conditions were trialed, as very poor or no amplification was seen for a number of samples, particularly high PVC. The different conditions trialed included different primer set (V9 region), varied primer concentrations (final concentration 500 nM per sample), choice of polymerase, input DNA concentration, annealing temperature gradient, and change in elongation time. The final amplification conditions were 98 °C for 1 min, followed by 10 cycles of denaturation at 98 °C for 10 s, annealing 44 °C for 30 s, and elongation 72 °C 15 s, then 20 cycles of 10 s denaturation at 98 °C, annealing 62 °C for 30 s and elongation at 72 °C for 15 s, and a final elongation step at 72 °C for 7 min. However, there were still samples which failed to amplify in these reactions. After amplification, PCR products from a single amplification were quantified on a Qubit Fluorometer 3.0 using a dsDNA high sensitivity assay kit (Thermo Fisher) and pooled at an equimolar concentration, before sequencing on a MiSeq Illumina sequencer (V300 paired-end) at the Ramaciotti Centre for Genomics (Sydney, Australia).

Demultiplexed raw reads for the 16S rRNA and 18S rRNA amplicons were quality controlled, adapters removed using cutadapt [39] and processed using DADA2 [40] using an optimized protocol to produce Amplicon Sequence Variants (ASVs). Briefly, the R1 and R2 terminal ends were trimmed to remove low-quality bases (for 16S rRNA, R1 = 260, R2 = 250; for 18S rRNA, R1 = 275, R2 = 255), in order to produce the highest number of merged reads following the error rate discovery and removing of chimera sequences. Taxonomy was assigned using the assignTaxonomy and assignSpecies implemented in DADA2 against the Silva database (v. 138) [41] for the 16S rRNA and Protist Ribosomal Reference (PR2) database (v 4.12) [42] for 18s rRNA. Amplicon data, as well as metagenomic raw data, were deposited to NCBI under bioproject PRJNA756323.

### Analysis of photosynthetic community members from 16S rRNA amplicon data

Bacterial ASVs assigned to cyanobacteria and chloroplasts were extracted and further classified to look in detail at the membership of the photosynthetic community. The ASVs assigned to plastids were taxonomically classified with the assignTaxonomy in DADA2 against the PR2 database (v. 4.12). Cyanobacteria sequences were classified using blastn against a curated database of cyanobacterial 16S rRNA sequences retrieved from Cyanorak Information system v2.1 [43]. Only those hits with a percentage identity of > 99% were retained.

### Sequencing, assembly, and functional characterization of metagenomes

Extracted DNA was used to generate metagenomic libraries, which were prepared at the Ramaciotti Center for Genomics (Sydney, Australia) using the Nextera DNA Flex library preparation kit (Illumina Inc.) for 16 samples and sequenced on a NovaSeq6000 (Illumina Inc.) on a 2 × 150-bp High Output run. Adapter and low-quality reads were removed using Trimmomatic [44]. Clean reads were then taxonomically classified with Kaiju [45]. Quality filtered reads were assembled into contigs using metaSpades [46] (using multiple Kmers up to K121). Contigs were concatenated, and open reading frames (ORFs) were identified with Prodigal (v. 2.6.1) in metagenome mode [47]. Genes were clustered at 95% identity with 60% length coverage with CD-HIT [48]. After dereplication at 95% identity, a set of 967,522 genes was obtained. Genes were then functionally classified using eggNOG (v.5) [49] which leverages the Kyoto Encyclopedia of Genes and Genomes (KEGG) and Cluster of Orthologous Genes (COG) databases. Of the total dereplicated genes, 48% had hits on either the KEGG or COG database. A gene count file was then generated mapping the quality-filtered reads against the gene catalog using BBMap (http://jgi.doe.gov/data-and-tools/bb-tools/).

### Metagenome binning

Contigs longer than 1000 bp were binned using metabat2 [50] in Anvi’o v. 6.2 [51]. Bins were then manually curated in Anvi’o, and the resulting metagenome-assembled genomes (MAGs) were quality checked with both the Anvi’o quality check for bins and CheckM [52]. Medium- and high-quality bins, with completion > 50% and contamination < 10%, were retained for further analysis. Each MAG was taxonomically classified using the GTDB-tk [53] program against v 1.4.1 of the GTDB database and functionally characterized using EnrichM v 0.5.0 (https://github.com/geronimp/enrichM). Predicted doubling time were calculated for each MAGs using the gRodon package in R [54]. The scripts “anvi-get-sequences-for-hmm-hits,” “--return-best-hit,” “--get-aa-sequence,” and “--concatenate” were used in Anvi’o to retrieve ribosomal protein from each MAGs. The proteins were then concatenated and aligned using MAFFT [55]. FastTree2 [56] was then used to construct the phylogenetic tree using the maximum likelihood method with 1000 bootstraps.

### Statistical analysis

Statistical analyses were performed in R (v. 4) [57]. An ANOVA followed by Tukey’s test was used to test the significance of differences observed between treatments at day 6 for cytometric counts, chlorophyll concentrations, photochemical efficiency, and trace metal concentration measurements. For the amplicon analysis, samples that had less than 3000 reads for either the 16S rRNA or 18S rRNA analysis were removed and not considered in statistical analyses. Dissimilarity between samples and treatments was visualized with a non-metric multidimensional scaling (nMDS) plot based on a Bray-Curtis dissimilarity matrix of the rarefied ASVs count for each phylogenetic marker. Significant effects of different treatments were evaluated with a multivariate PERMANOVA using the Adonis2 function in Vegan [58]. The DESeq2 [59] package was used to identify ASVs that significantly contributed to the observed community dissimilarity between the treatments and the control after 6 days of exposure.

For metagenomic analyses, gene counts were normalized using the reads per kilobase of transcript per million of mapped reads (RPKM) value that considers both the gene length and the total number of reads per sample. To calculate the enrichment of COG categories for each treatment compared to the control, normalized gene counts were aggregated to the COG categories to which they belonged, and the count was transformed to relative abundance. Relative abundance of each COG category per treatment was then pairwise compared to the SW control to determine the percentage of enrichment, and enrichment significance was evaluated with ANOVA followed by a pairwise *t*-test with the *p*-value false discovery rate (FDR) corrected. The DESeq2 package was then used to evaluate the differential abundance of assigned KEGG Orthology identifiers (KOs) for each treatment compared to the SW control. Only results with a log fold change > 2 or < − 2 and a corrected *p*-value < 0.01 were then considered for further analysis.

## Results and discussion

### Photosynthetic communities respond negatively to PVC leachate and zinc exposure

**Fig. 1:**
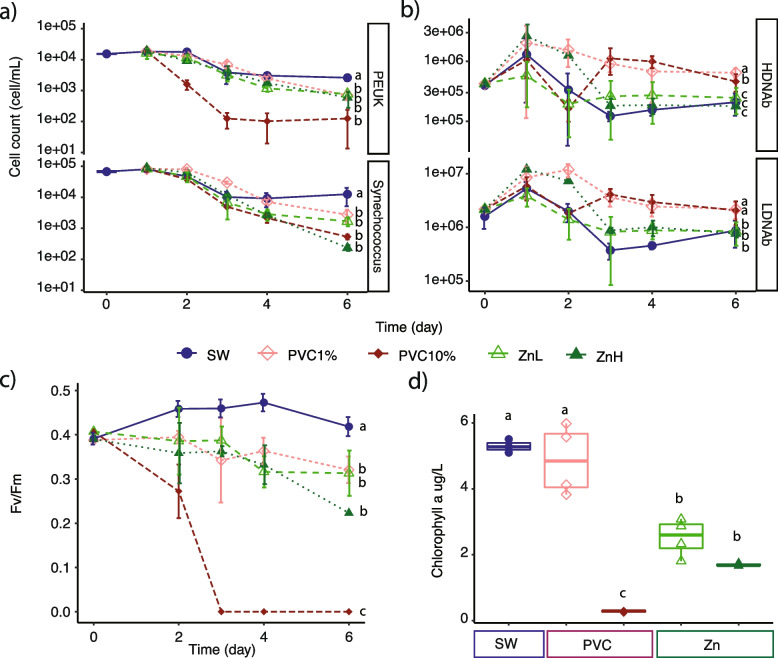
Impact of plastic leachate and zinc on phytoplankton and bacteria over the 6-day experiment. Cell counts of **a ***Synechococcus* and photosynthetic picoeukaryote (PEUK) populations and **b** high-DNA bacteria (HDNAb) and low-DNA bacteria (LDNAb) populations throughout the 6-day experiment. Data points represent flow cytometric cell counts derived from the mean values of the quadruplicate biologically independent samples (except for high zinc (ZnH), for which just 2 replicates are available). The lowercase letters identify which treatments were found to be statistically significantly different (*p* < 0.01) at the final time point (6 days of exposure). Impact of PVC plastic leachate and zinc treatments on **c** photosynthetic quantum yield (*F*_*v*_/*F*_*m*_) during the 6-day experiment and **d** final chlorophyll a concentration (after 6 days of exposure). Data points are the average for quadruplicate independent biological samples (except for ZnH, where *n* = 2). For 10% PVC samples, there was no detectable photosynthetic capacity after day 2. Error bars on each point represent the standard deviation. PVC1, 1% PVC leachate; PVC10, 10% PVC leachate; ZnL, low zinc; ZnH, high zinc

To investigate the impact of PVC leachate and zinc, a common plastic additive, on marine microbial communities, we measured the abundance of different photosynthetic and heterotrophic microbes and viruses daily for 6 days following exposure (Fig. 1, Fig. S4, Additional file 2: Tables S1, S2). Photosynthetic communities were severely impacted by PVC leachate (Fig. 1a, Fig. S4a, *p* < 0.01), with the cell abundances of the ubiquitous cyanobacteria *Synechococcus* decreasing by 5.5-fold in the 1% PVC treatments, and by up to 30-fold in the 10% PVC treatments compare to the control by day 6 (*p* < 0.02, Fig. 1a, Additional file 2: Table S3). In addition, photosynthetic picoeukaryotes (PEUK) abundances declined by 4-fold and 16-fold in the 1% PVC and 10% PVC treatments, respectively (*p* < 0.001, Fig. 1a, Additional file 2: Table S3). These results reinforce previous findings on the deleterious effect of PVC leachate exposure on photosynthetic prokaryotes in culture [26, 27]. Following exposure to zinc, the *Synechococcus* population also decreased rapidly, with a 9-fold reduction for low zinc treatment (ZnL) and a 60-fold reduction in the high zinc treatment (ZnH) (*p* < 0.02, Fig. 1a, Additional file 2: Table S3). While still decreasing significantly, the abundance of picoeukaryotes was less impacted by zinc, with 3.6- and 4.6-fold decreases observed in the ZnL and ZnH treatments, respectively (*p* < 0.001, Fig. 1a, Additional file 2: Table S3). Although our understanding of zinc toxicity in phytoplankton is still limited, recent reports indicate larger cyanobacteria, and photosynthetic eukaryotes tend to be much more tolerant of such exposure than picocyanobacteria [29, 60, 61]. Indeed, some picocyanobacterial strains suffer significant declines in growth and photosynthesis when exposed to Zn concentrations as low as 27 μg/L [29, 60, 61]. Tolerance to zinc toxicity likely depends on the availability of defense mechanisms such as organic or inorganic precipitation, biotransformation, active transport, and sequestration. Genes encoding these protective mechanisms are heterogeneously distributed among cyanobacteria [62]. Zinc is also usually considered a limiting nutrient for photosynthetic eukaryotes, and it is possible that the addition of Zn within some concentration range may enhance their growth [60].

Larger photosynthetic eukaryotes were severely impacted by the 10% PVC treatment, with a 2.4-fold decrease in counts recorded for NEUK and a 9.5-fold decrease recorded for PE-EUK (*p* < 0.05, Fig. S4a, Additional file 2: Table S3). However, neither the 1% PVC nor the two zinc treatments significantly affected NEUK and PE-EUK compared to the control after 6 days of incubation. These results suggest phytoplankton response to PVC leachate is species-specific and that larger phytoplankton appears less impacted by low concentrations of plastic leachate. Past work looking at the impact of other contaminants on different phytoplankton suggested that larger cells may have a reduced likelihood of incorporating contaminants due to their smaller surface area to volume ratio [63]. Our findings are also in accordance with recent studies reporting different degrees of growth inhibition on model photosynthetic organisms when exposed to different concentrations of PVC leachate [17] and PVC particles [64, 65].

Consistent with these declines in photosynthetic cell abundances in leachate treatments, we observed sharp reductions in photosynthetic efficiency (*F*_*v*_/*F*_*m*_) for these communities, with no measurable *F*_*v*_/*F*_*m*_ signal registered for 10% PVC by day 3 and a 1.5-fold reduction for the 1% PVC by day 6 (Fig. 1c, Additional file 2: Table S4). We also recorded a 5-fold reduction in chlorophyll concentration in the 10% PVC treatment (*p* < 0.01) and ~ 3-fold reduction in the ZnH treatment (*p* < 0.01) (Fig. 1d). These results reveal that PVC leachate strongly affects the abundance, physiology, and composition of photosynthetic marine microbial assemblages. These findings are similar to those of other studies that have examined the impacts of leachates from other plastic types on algal photosynthesis. Specifically, *Chlorella vulgaris F*_*v*_/*F*_*m*_ declined during exposure to leachate from polyurethane foam [66], and a recent work on the chlorophyte *Scenedesmus vacuolatus* showed adverse effects on photosynthesis following exposure to leachates derived from UV-irradiated polyethylene [67].

In direct contrast to the phototrophic microbes, heterotrophic bacterial populations displayed on average a 2.5-fold increase in cell abundance within the PVC treatments by day 6 (*p* < 0.01, Fig. 1b) with both the HDNA and LDNA bacteria showing a significant increase in cell counts compared to the seawater control. This result is in accordance with the findings of previous studies indicating that some heterotrophic bacteria can utilize the organic matter dissolved from certain plastics to enhance their growth, based on work using leachate from new low-density polyethylene (LDPE) [16], UV-irradiated post-consumer polystyrene and polypropylene [68], and more recently with mixed aged plastics [69]. Interestingly, while LDNA bacterial counts were similarly enriched in both PVC treatments compared to the control, for HDNA bacteria, the 1% PVC leachate exposure resulted in significantly higher increases than the 10% PVC treatment (*p* < 0.01, Fig. 1b), suggesting that certain members of the heterotroph population respond differently depending on the concentration of leachate they are exposed to. It is not clear whether these effects are linked to specific inorganic or organic leachate components, but previous work has shown that the cell membranes of high-nucleic acid content bacteria are damaged much faster than those of low-nucleic acid bacteria when exposed to chlorine dioxide and permanganate [70], suggesting that this group might be more susceptible to damage from PVC leach components. Additions of zinc alone did not have a significant effect on the abundance of heterotrophic bacteria (*p* < 0.01; Fig. 1b, Additional file 2: Table S3). This finding supports the hypothesis that utilization of organic components from the PVC leach is a significant driver of the observed heterotrophic increases, rather than this simply representing stimulation due to dying phytoplankton providing a source of dissolved organic carbon, as zinc treatments resulted in declining phytoplankton populations which were not coupled with changes in heterotroph abundance.

Viral populations were also monitored across the experiment using flow cytometry. Five separate sub-populations of viruses were identified, and all showed a significant decline in abundance in the high PVC treatments compared to the control by day 6 (*p* < 0.01, Fig. S4d, Additional file 2: Tables S2, S3), which could potentially be linked to shifts in the composition of the microbial communities. However, to our knowledge, no other plastic leachate toxicity work has looked at the impacts on virus populations; it is therefore not possible to speculate how viral populations might be affected by leach from other plastics.

The concentration of specific inorganic ions within the PVC leachates, particularly heavy metals known to inhibit photosynthesis in algae and plants [71], was measured in seawater control and PVC treatment samples after the 6-day incubation (Table 1, Additional file 2: Table S5a). Zinc was the most enriched element in PVC leachate, with concentrations 20-fold and 180-fold higher than the control for 1% PVC and 10% PVC respectively (*p* < 0.01, 0.4 ± 0.06 mg/L for 1% PVC and 3.6 ± 0.15 mg/L for 10% PVC). This is similar to what has been reported previously using the same PVC product [26, 27] and what has been seen with different PVC items (avg 6.357 mg/L in marine media) [17]. Based on these measurements, the concentration in the ZnL treatment was 3.4-fold higher than 1% PVC (but 2.6-fold lower than 10% PVC), while the concentration in the ZnH treatment was 3.6-fold higher than the 10% PVC treatment. Cobalt, nickel, and copper, which are also known to be detrimental at high concentrations for photosynthetic organisms [72], were also detected in the 10% PVC treatment (Co 3.08 ± 0.04 μg/L, Cu 0.75 ± 0.15 μg/L, Ni 1.75 ± 0.13 μg/L) but were below detection limits in all other treatments. Given that the 10% PVC treatment had a more detrimental impact on photosynthetic microorganisms than the ZnH treatment (lower photosynthetic cell counts, *F*_*v*_/*F*_*m*_ and chlorophyll concentrations), we conclude that zinc only partially contributed to these negative impacts. Other contributors to the negative impacts of PVC leachate on photosynthetic microorganisms are likely the additional heavy metals, along with organic components leached from PVC, such as phthalates [18], which have previously been associated with photosynthetic impairment in plants and algae [65, 73, 74].

**Table 1:** Concentrations of metals in controls and PVC treatments, determined via ICP-MS (*n* = 3) at the beginning of the experiment (day 0) and after 6 days of incubation (day 6). The average concentrations are included plus or minus the standard error. Concentrations for the Zn treatments were not included as for these only Zn was measured and was ZnL 0.13 mg/L and ZnH 1.3 mg/L. (*) indicates treatments for which the metal concentration was just above the limit of the detection in one of the samples tested. Bold text indicates metals that were significantly enriched in the 1% and 10% PVC treatments compared to the SW control (pairwise t-test, *p* < 0.05). For those elements (cobalt and nickel) that were present at detectable levels in the undiluted PVC and 10% PVC but were below the minimum detection limits (MDL) for the SW samples, we used the MDL values for calculations of statistically significant enrichment

	**Day 0**		**Day 6**		
	PVC leachate	SW control	SW control	PVC 1%	PVC 10%
**As (μg/L)**	0.6*	1.93 ± 0.25	1.78 ± 0.11	1.62 ± 0.19	1.47 ± 0.37
**Ba (μg/L)**	2.76 ± 0.06	4.59 ± 0.13	4.45 ± 0.13	4.79 ± 0.15	4.34 ± 0.11
**Co (μg/L)**	30.81 ± 0.3	-	-	-	**3.08 ± 0.049**
**Cu (μg/L)**	3.91 ± 0.07	0.68*	0.54*	-	0.75 ± 0.15
**Fe (μg/L)**	5.91 ± 0.21	6.27 ± 0.33	6 ± 0.66	6.33 ± 0.35	6.72 ± 0.84
**Mo (μg/L)**	0.54 ± 0.15	15.27 ± 0.35	15.28 ± 0.29	14.99 ± 0.21	13.89 ± 0.46
**Ni (μg/L)**	16.06 ± 0.69	-	-	-	**1.75 ± 0.13**
**Se (μg/L)**	0.52 ± 0.017	-	0.56*	0.7*	-
**U (μg/L)**	0.228 ± 0.02	3.75 ± 0.08	3.68 ± 0.10	3.68 ± 0.049	3.34 ± 0.021
**V (μg/L)**	-	1.51 ± 0.19	1.31 ± 0.21	-	0.91 ± 0.18
**Zn (μg/L)**	3303 ± 46.54	2.79 ± 0.49	1.95 ± 0.53	**40.41 ± 6.08**	**360.31 ± 15.51**

### PVC leachate and zinc select for different bacterial and eukaryotic communities

**Fig. 2:**
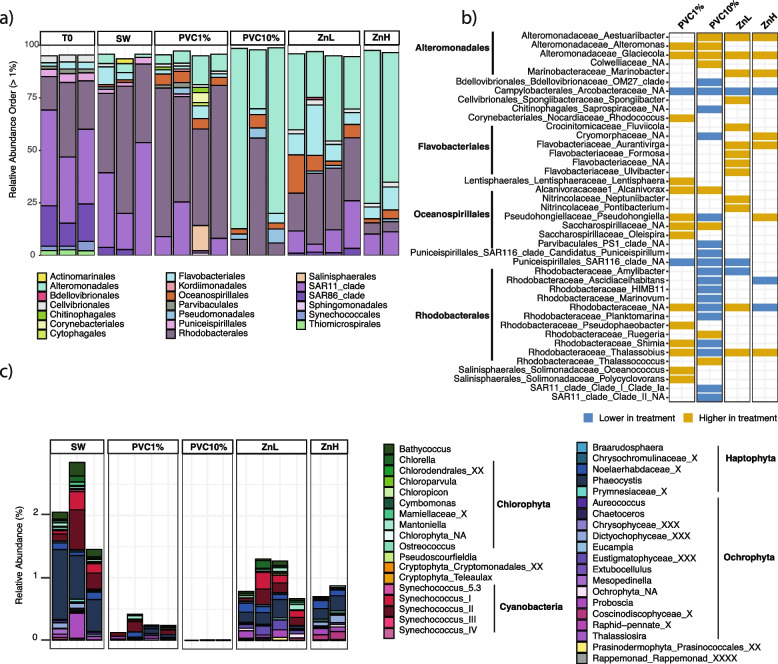
Comparison of bacterial community composition across PVC leachate and zinc treatments. **a** Stacked bar plot showing the relative abundance of bacterial orders which contribute > 1% relative abundance based on 16S rRNA amplicon data. **b** The set of bacterial genera observed to be significantly different between each treatment compared to the control using DESeq2 analysis (*a* = 0.01, *p* < 0.01), which contribute > 1% relative abundance. Shading indicates whether genera were significantly more abundant in the treatment or in the control (blue: lower in the treatment; orange: higher in the treatment). **c** Stacked bar plot indicating the relative abundance of photosynthetic community members in each sample, based on the analysis of cyanobacteria and plastid sequences from the 16S rRNA amplicon sequence analysis at day 6. For the 10% PVC treatment, only two replicates are shown as the third replicate for this treatment did not contain any cyanobacteria or plastid sequences

**Fig. 3:**
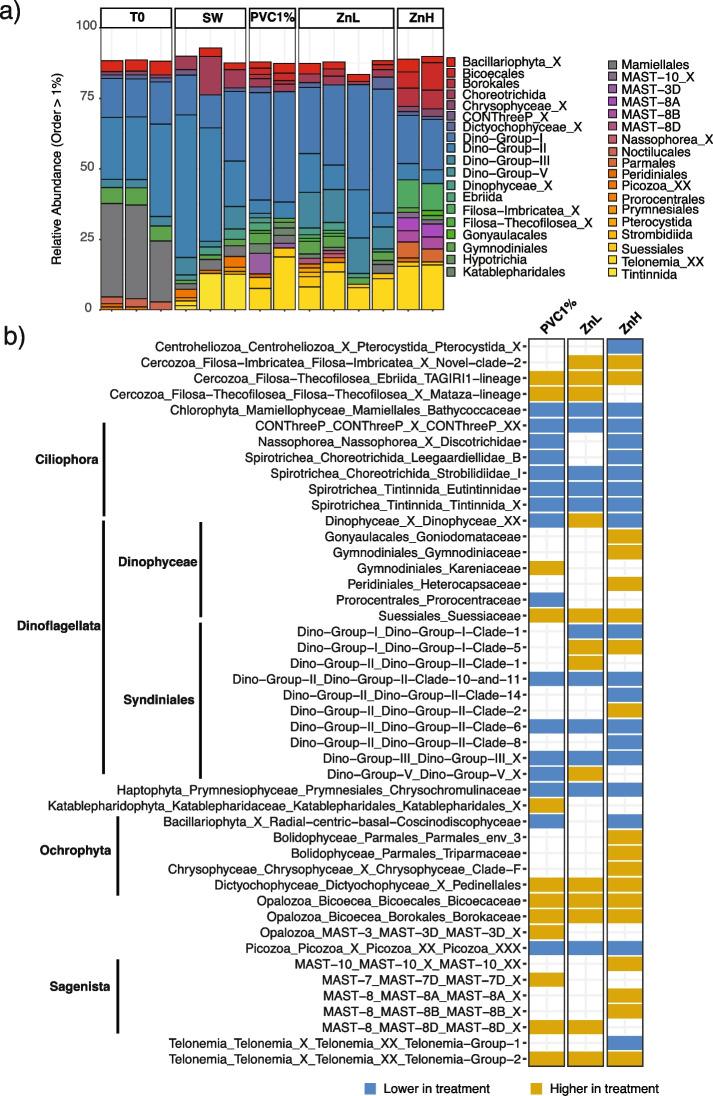
Comparison of eukaryotic community composition across PVC leachate and zinc treatments. **a** Stacked bar plots of the relative abundance of eukaryotic order which contributed > 1% relative abundance based on 18S rRNA amplicon data. **b** The set of eukaryotic genera observed to be significantly different between treatments and controls using DESeq2 analysis (*a* = 0.01, *p* < 0.01) which contribute > 1% relative abundance. Shading indicates genera which were statistically different in abundance in the treatment compared to the control (blue: lower in the treatment; orange: higher in the treatment)

To investigate which microbial taxa were impacted by PVC leachate and zinc, we performed amplicon sequencing (16S rRNA and 18S rRNA genes) and shotgun metagenomics. After 6 days of incubation, microbial community composition was significantly different for each treatment relative to the control (Figs. 2a and 3a, Fig. S5e, f), with community profiles grouping by treatment (PERMANOVA *p* < 0.001, Fig. S5c, d, Additional file 2: Table S6). Consistent with previous studies on plastisphere communities [11, 75], there was a significant decline in alpha diversity (Shannon diversity and richness) of the prokaryotic community exposed to 10% PVC leachate (Fig. S5a, Additional file 2: Table S7). The alpha diversity indexes of the eukaryotic communities were not significantly different among any of the treatments (Fig. S5b, Additional file 2: Table S7). The communities experienced changes in the relative abundance of some bacterial groups during the course of the experiment (from T0 to SW on day 6 in Fig. S5), likely due to the bottle effect [76, 77]. However, these changes were significantly less pronounced than the effects of the leachate and Zn treatments, indicating that the observed community shifts were primary driven by these stressors.

The relative abundance of globally distributed SAR11 and Rhodobacterales declined in all treatments (Fig. 2a). These two taxa dominated the seawater samples, and their combined relative abundance was greater than 70%, which is consistent with their typical abundance at this sampling site [28, 78], and in the marine environment in general [13, 79, 80] (Fig. 2a). In comparison, the proportion of SAR11 decreased to less than 1% relative abundance in 10% PVC, while the Rhodobacterales decreased by 1.5-fold in the 10% PVC and by 8.8-fold in the ZnH treatment. While the relative abundance of SAR11 is usually quite low within the plastisphere microbiome compared to the surrounding seawater [10, 11], Rhodobacterales can account for a large fraction of the bacterial community on plastic particles [11, 81]. Analyses at the genus and amplicon sequence variant (ASV) level identified specific SAR11 and Rhodobacterales groups that were most significantly impacted (Fig. S6, Additional file 2: Table S8). These included SAR11 clade Ia and the Rhodobacterales *Amylibacter*, *Ascidiaceihabitans*, and *Planktomarina* genera, which significantly declined in the 10% PVC treatment (*p* < 0.01; Fig. 2b, Fig. S6, Additional file 2: Table S8). The decline of SAR11 and Rhodobacteraceae may have multiple explanations. Many genera belonging to the Rhodobacterales are known to establish mutualistic relationships with phytoplankton [80], and their decline could have been caused by the collapse in the abundance of photosynthetic microorganisms. The dying phototrophs in PVC leachate and Zn treatments may also have supplied some particulate and dissolved organic matter that would enable copiotrophs to outcompete the oligotrophic microbes, thus resulting in a lower relative abundance of oligotrophs in the treatments. However, the reduction in SAR11 and other oligotrophs may not be solely to being outcompeted by copiotrophs, as there were more significant losses in both PVC treatments compared with the Zn treatments. This points to the possibility of specific organic chemicals leaching from PVC plastic negatively affecting the oligotrophs. For example, SAR11 clades, adapted to thrive in generally stable and low-nutrient environments, are characterized by small and streamlined genomes [13, 82] that may render these organisms less tolerant to exposure to complex chemical mixtures, perhaps due to reduced transporter suites or metabolic capabilities.

The overall abundance of photosynthetic groups was relatively low (1.5–2.8% in SW; based on cyanobacteria and plastid sequences from the 16S rRNA); however, the detrimental impact of the PVC treatments was still evident in this data (Fig. 2c). The relative abundance of photosynthetic organisms dropped to < 0.4% in the 1% PVC treatment (*p* < 0.05) and < 0.001% in the 10% (*p* < 0.01) PVC treatment (Additional file 2: Table S9). Zinc treatments resulted in less substantial declines, with photosynthetic organisms making up ~ 1% in ZnL and ~ 0.75% in ZnH. Consistent with the flow cytometric counts, key photosynthetic groups significantly declined in relative abundance (*p* < 0.01, Additional file 2: Table S9) in the PVC and ZnH treatments, including *Synechococcus* (~ 200-fold decrease in PVC 10%) and the Chlorophyta picophytoplankton *Bathycoccus* and *Ostreococcus* (no longer detectable in both the 10% PVC and ZnH treatments). These results provide further support that components of the PVC leachate other than Zn may be responsible for the majority of the decline in photosynthetic community members. While the detrimental effect of PVC leachate has previously been documented in *Prochlorococcus* cultures [26, 27], our results are the first to show that PVC leachate negatively impacts communities of important primary producers as well as some major heterotrophic bacterial groups that are key players in several oceanic biogeochemical cycles. Marine phytoplankton are at the base of the marine food web and are therefore essential for the health and functioning of marine ecosystems. Changes in the abundance, productivity, and composition of these communities in response to plastic leachate exposure therefore have the potential to impact higher trophic levels that are intrinsically dependant on phytoplankton.

In contrast to SAR11, some Rhodobacterales and phototrophic microbes, certain heterotrophic bacterial groups benefited from exposure to PVC leachate and added zinc. Among these, the Alteromonadales increased dramatically from a relative abundance of 4% in the control to an average of 64% of the total relative abundance across the 10% PVC treatments and up to 65% relative abundance for the ZnH samples (*p* < 0.01, Fig. 2a, b, Additional file 2: Table S8). This was due largely to shifts in the relative abundance of a small number of specific genera and ASVs, with *Glaciecola* and *Alteromonas* contributing to a large proportion of the increased relative abundance of this order in the PVC and zinc treatments (Fig. 2b, Fig. S6). Notably, the relative abundance of a single *Alteromonas* ASV (ASV_9) ranged from 5 to 37% in the 10% PVC treatment, while its relative abundance in the control was below 0.1%. Members of the Alteromonadales have been identified in plastisphere communities in previous studies [10, 83, 84], with multiple genera capable of degrading hydrocarbons [85] and high molecular weight dissolved organic matter [85]. PVC treatments specifically resulted in a significant increase in bacterial genera that are known to be able to degrade phthalates, including *Alcarnivorax* [86] and the Rhodobacteraceae genus *Thalassococcus* [87, 88]. In contrast, the relative abundance of the Flavobacteriales increased by 2.5-fold and 3.8-fold in the ZnH and ZnL treatments, respectively. The increase in this bacterial order might be linked to changes in the eukaryotic community, as these organisms are usually associated with blooms of specific diatom species [89]. These results revealed that PVC leachates, and zinc addition to a lesser extent, caused a shift from Alphaproteobacteria to Gammaproteobacteria-dominated communities (Fig. S5e), inducing a major restructuring of planktonic heterotrophic bacterial assemblages.

Eukaryotic communities also exhibited some dramatic changes (identified through 18S rRNA gene amplicon analysis), evident from the phylum level down, for both photosynthetic and heterotrophic eukaryotes. In keeping with the flow cytometry and 16S rRNA (plastid) relative abundance results, the Mamiellales order, comprising multiple species of photosynthetic picoeukaryotes including *Bathycoccus* species, declined significantly in all treatments (*p* < 0.01, Fig. 3a, b, Additional file 2: Table S10). Some Ochrophyta (Parmales and Chrysophyceae families), however, increased in relative abundance following zinc addition, possibly due to release from zinc limitation [90] (Fig. 3, Fig. S7). In addition, some mixotrophic dinoflagellate orders (e.g., Gonyaulacales, Gymnodiniales, and Peridiniales) significantly increased in 1% PVC and zinc treatments. This is of potential wider concern, as all of these dinoflagellate orders harbor species that produce toxins and can cause harmful algal events [91, 92]. Some of these harmful algae have previously been found associated with floating plastic debris [10, 11, 93, 94]. Heterotrophic eukaryotes were also impacted by the PVC leachate and zinc treatments, with significant decreases in the relative abundance of numerous genera within the Spirotrichea class (*p* < 0.01, Fig. 3b, Additional file 2: Table S10). Telonemia, in contrast, contributed less than 1% in relative abundance in the controls but between 5 and 15% in all the treatments (*p* < 0.01, Fig. 3b, Additional file 2: Table S10). Notably, most of the orders with species known to graze upon *Synechococcus* and picoeukaryotes, such as Tintinnida and Choreotrichida (Spirotrichea) [95], and some Dinophyceae, were significantly impacted by exposure to 1% PVC, based on 18S rRNA relative abundance data. This result supports that the strong decline experienced by photosynthetic community members in both the 1% and 10% PVC treatments is mostly due to the toxicity of the leachate, rather than grazing pressure. Relatively few plastisphere studies have included 18S rRNA surveys, and, to our knowledge, this is the first time that the impact of plastic leachate exposure has been reported for a heterotrophic eukaryotic community. Our results demonstrate that plastic leachates can elicit either positive or negative effects on certain members of these communities, highlighting the need to consider unicellular eukaryotes in the future analysis of plastic leachate effects.

### PVC leachate and zinc exposure impact the functional potential of marine microbial populations

**Fig. 4:**
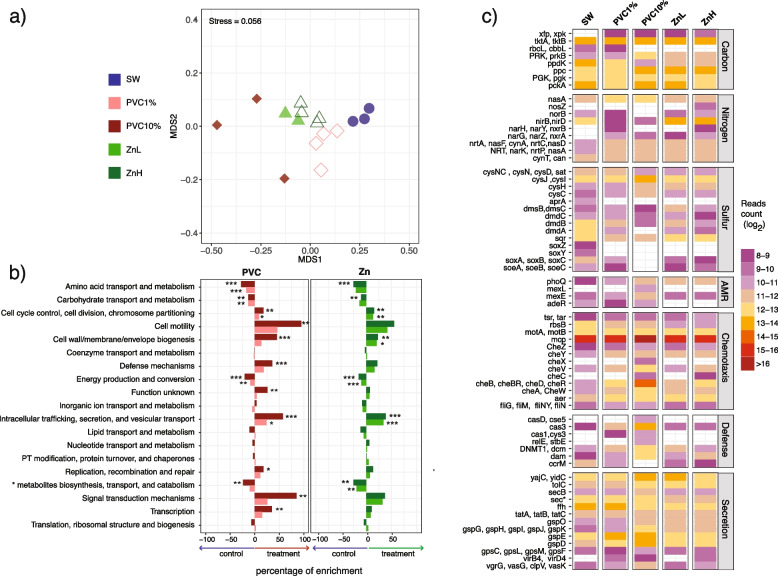
Plastic leachate and zinc impact on the functional potential of marine communities. Distribution of microbial functional genes annotated with KEGG and/or COG retrieved from metagenomic analyses of all samples. **a** Multidimensional scaling plot (nMDS) of the Bray-Curtis dissimilarity based on the normalized distribution of functional genes. Stress of the nMDS is displayed on the plot. **b** Relative percent enrichment and significance of the different COG categories in each treatment compared to the control. The mean relative abundances of COG categories were summed for each treatment. Categories with less than 1000 reads mapping back were excluded from the plot. Bars are color-coded as indicated in **a**. Asterisks (*) indicate significant enrichment relative to the seawater control based on the pairwise *t*-test (FDR-corrected) calculated on COG category relative abundances (*p < 0.05, **p < 0.01, ***p < 0.001) (*metabolites = secondary metabolites). **c** Heatmap indicating the log_2_ of DESeq2-normalized read count observed in the control and each treatment for differentially abundant genes that belong to the KEGG pathways related to important COG categories. The set of genes displayed are those that had > 500 reads mapping back across the whole dataset and a DESeq2-derived log_2_-fold change of read counts > 2 and < − 2. (sec*, secA,D,E,F,G,Y; AMR, antimicrobial resistance)

To understand how exposure to PVC leachate or zinc impacted microbial community functional potential, we carried out shotgun metagenomic analyses. Consistent with the 16S rRNA and 18S rRNA amplicon data, the functional profiles within PVC leachate and zinc treatments were distinct from the control, with all treatments except the 10% PVC treatment showing tight clustering of samples (nMDS; Fig. 4a). Taxonomic assignment of reads showed that most of the genes (> 97%) in the metagenome belonged to bacteria (Additional file 2: Table S11). The small eukaryotic component (1.6%) dropped dramatically to 0.14% in the 10% PVC treatment (Additional file 2: Table S11) further indicating the sensitivity of eukaryotic microorganisms to high levels of PVC leachate.

Within the set of annotated genes, similar COG categories were enriched in each of the treatments relative to the control (Fig. 4b). Communities exposed to PVC and zinc treatments were all significantly enriched in genes related to intracellular trafficking, secretion, and cell cycle processes, while the 10% PVC treatment additionally resulted in significant increases in genes involved in cell motility, defense mechanisms and signal transduction. Enrichment in the latter set of COGs may be important in enabling rapid adaptations to changes in environmental conditions and have previously been linked with a copiotrophic lifestyle [96]. In contrast, genes assigned to amino acid and carbohydrate transport and metabolism, energy metabolism, and secondary metabolites biosynthesis, which are COGs generally associated with an oligotrophic lifestyle [96], significantly decreased in relative abundance for all the treatments compared to the control (Fig. 4b). This indicates a clear shift in the metabolic potential of microbial communities exposed to zinc and PVC leachate.

To further investigate these metabolic shifts, genes were assigned to 6345 main functional orthologs (KO; KEGG Orthology Database). Relative to the control, 702 genes were significantly different in abundance in the 10% PVC treatment, while 384 were different in the ZnH treatment (Additional file 2: Tables S12, S13, S14, S15). For both the PVC leachate and zinc treatments, there were significantly fewer genes associated with carbon fixation, especially for photosynthesis. Specifically, there was a 46-fold decline in genes mapping to the main RuBisCO gene *rbcL* in the 10% PVC treatment compared to the control (Fig. 4c), which is consistent with both the photophysiology measurements and amplicon sequencing data.

Chemotaxis, biofilm formation, and secretion genes were highly enriched in all treatments (increases of ~ 10-fold in most cases) relative to the control. This pattern was particularly notable in the 10% PVC treatment, where the relative abundance of specific chemotaxis genes was 400-fold higher than in the control (Fig. 4c). The higher diversity of Che-like chemotactic receptor domains in the 10% PVC may provide the microbes enriched in this treatment with a better capacity to detect chemical gradients. Given that hydrocarbons can act as chemoattractants for various microorganisms [97, 98], PVC leachate may create hotspots of newly available organic matter that favor motile and chemotactic bacteria. For some pathogenic bacteria, chemotactic genes also control bacterial adhesion and hence the potential for biofilm formation [99]. In addition to the organic chemicals leached from the PVC, the collapse of phototrophic microorganisms, particularly in the 10% PVC samples (Fig. 1a, Additional file 2: Table S3), may have provided additional organic matter and surface from cell debris for copiotrophic growth.

Genes involved in protein secretion, DNA transfer, and defense mechanisms were also enriched in all the treatments, with a notable increase in type IV secretion system genes (Fig. 4c). Type IV secretion systems, which are typically associated with DNA exchange and pathogenicity, have been observed in plastisphere communities in the past [84] and could indicate an increased potential for horizonal gene transfer. DNA exchange has been linked to an increase in antimicrobial resistance genes in the plastisphere, and we found several antimicrobial resistance genes enriched in the 10% PVC (Fig. 4c). Observing an increase in secretion system and antibiotic resistance genes in communities exposed to leached chemicals, rather than directly on plastics, indicates plastic-impacted marine communities may be even more likely to show traits associated with pathogenicity than previously realized.

### Metagenome-assembled genomes from plastic and zinc treatments encode diverse xenobiotic degradation pathways

**Fig. 5:**
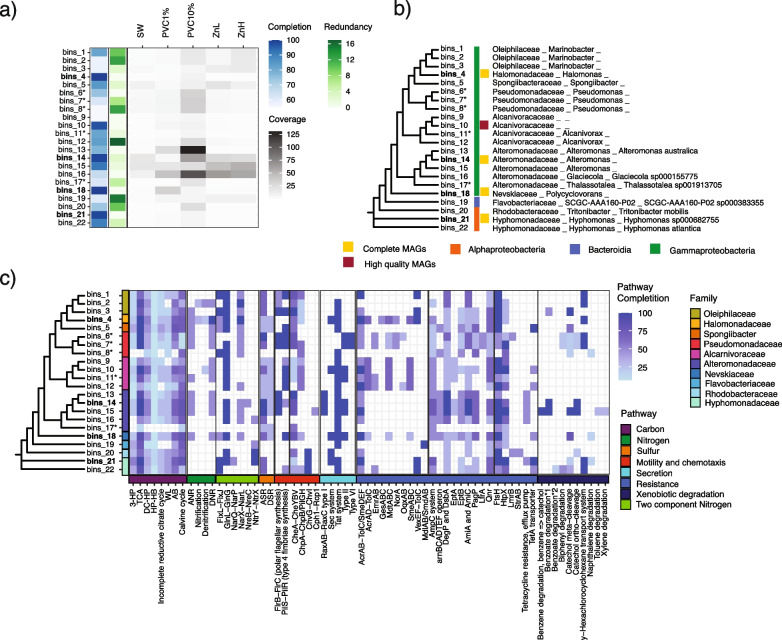
The main characteristics and functional annotations of the 22 identified MAGs recovered from the metagenomes. **a** Heatmap indicating the percentage completion, redundancy, and coverage of each MAG in each treatment. Coverage for each MAG was calculated in Anvi’o v. 6.2. Bold bin names indicate complete MAGs. An asterisk (*) indicates MAGs that were significantly more abundant in a PVC treatment compared to the control (*p* < 0.001, FDR-corrected). **b** Phylogenetic tree of the curated MAGs, based on MAFFT alignment of recovered ribosomal marker sequences. Complete and high-quality MAGs (completion > 97%, redundancy < 3%) are color-coded along with the taxonomic class to which each MAG is assigned. **c** The main complete or near-complete gene pathways of the selected biochemical functions for each MAG. Biochemical pathway completeness is indicated by blue shading. 3HP, 3-hydroxypropionate cycle; TCA, tricarboxylic acid cycle; DC/HB, dicarboxylate/4-hydroxybutyrate cycle; HP/HB, 3-hydroxypropionate/4-hydroxybutyrate cycle; WL, Wood-Ljungdah pathway; AB, Arnon-Buchanan cycle; ANR, assimilatory nitrate reduction; DNR, dissimilatory nitrate reduction; ASR, assimilatory sulfur reduction; DSR, dissimilatory sulfur reduction; benzoate degradation*1, benzoate degradation I (aerobic); benzoate degradation*2, benzoate degradation II (aerobic and anaerobic)

Co-assembly and binning of the 16 metagenomes yielded a total of 22 medium to high-quality metagenome-assembled genomes (MAGs) (completion > 50% and redundancy < 16%), which were differentially distributed across treatments (Fig. 5a, Additional file 2: Table S16). These MAGs belong to the Alphaproteobacteria, Gammaproteobacteria, and Bacteroidia classes and represent ten different bacterial families (Fig. 5b, Additional file 2: Table S16), all of which were observed at higher abundance in at least one treatment relative to the control (Fig. 5a). Twelve MAGs, including the *Alteromonas*, *Pseudomonas*, and *Tritonibacter* MAGs, were observed at higher abundance in PVC, with five of those significantly more abundant (*p* < 0.01) in PVC treatments compared to zinc treatments or the control (bins6-8 belonging to *Pseudomonas*, bins11 *Alcarnovorax*, and bins17 *Thalassotalea*) (Additional file 2: Table S16). The *Alteromonas* MAG bins_14 had a 99% match to *Alteromonas* ASV_9, which contributed an average of 20% relative abundance in the 10% PVC treatments. These leachate-enriched MAGs show gene repertoires indicative of extensive metabolic capabilities (Fig. 5c).

Consistent with the results of functional potential at the community level, the leachate-enriched MAGs encode pathways usually associated with copiotrophic and motile lifestyles. The *Alteromonas* and two of the *Pseudomonas* MAGs present a complete set of flagella biosynthesis genes, while the *Polycyclovorans* and Alcarnivoraceae MAGs possessed the complete set of genes required for fimbriae biosynthesis (Fig. 5c). All were enriched with a variety of chemotaxis and resistance-associated genes, such as antimicrobial resistance and efflux pumps (Fig. 5c). Additionally, most of these MAGs with high coverage in PVC samples had relatively fast predicted doubling times, between 3 and 9 h (Fig. S8, based on methods of Weissman et al. [54]), also consistent with these being copiotrophic organisms. These findings highlight the potential of PVC leachate to positively select for organisms that have the ability to quickly respond to changing environmental conditions.

Most of the MAGs with higher coverage in the PVC samples were also found to encode pathways associated with xenobiotic degradation (Fig. 5c). The gamma-hexachlorocyclohexane (g-HCH) transport system module, significantly enriched in PVC (Additional file 2: Table S17), has been associated with the capacity to transport lindane [100, 101], a highly toxic compound used in pesticides, and also found in plastic leachate [102]. Most of the *Alteromonas* and *Pseudomonas* MAGs were found to encode an almost complete catechol-ortho cleavage pathway, one of the main pathways involved in the biodegradation of phthalates [103], suggesting that these bacteria have the potential to break down and utilize organic components leached from the PVC.

## Conclusions

Current trends indicate that mismanaged plastic waste will increase up to 10-fold over the next 10 years [5, 8], suggesting that the marine plastic burden and its ecological impact will continue to escalate. Plastisphere research has indicated the potential for specific groups of microbes, including pathogens, to benefit from the new niches provided by plastic debris. Our findings reveal that plastic pollution has the potential to have an even greater influence on marine microbial communities than currently thought, due to chemical leaching. While changes in microbial communities associated with the plastisphere are largely restricted to particle surfaces, shifts among the planktonic community in response to chemicals that are released into the surrounding water column have the potential to exert wider impacts. Leachate-related plastic pollution impacts could extend over time, as we have shown previously that such PVC matting continues to leach substances capable of impairing the growth and photosynthesis of marine bacteria *Prochlorococcus* after 3 months of environmental weathering [27]. While this work reveals the capacity for chemicals leached from plastic debris to impact the structure and function of marine microbial communities, we note that these experiments were conducted using a methodology based on previous leachate ecotoxicology work [26, 32], rather than setting out to mimic explicit environmental exposure scenarios. Environmental impacts are likely to be highly variable, given that plastic pollution levels are very patchy and the precise makeup of such debris, in terms of leachates, is very heterogeneous [19]. Surveys of marine plastic conducted to date have covered a relatively small area of the world’s oceans and much work is still needed to understand how leachates from different plastic items vary in composition and leaching dynamics [104]. In situ oceanographic surveys of microbial communities in areas with contrasting levels of plastic pollution, in addition to further microcosm and mesocosm experiments involving different starting communities and leachates from a wider range of plastics will help in gaining a mechanistic understanding of this growing issue.

Here, we show that exposure to plastic leachate from PVC, a polymer used in many common plastic items, negatively impacts microbial picophytoplankton, both prokaryotic and picoeukaryotic, as well as the globally distributed SAR11 and members of the Rhodobacterales order, heterotrophic bacteria critical to the main marine biogeochemical cycles. While certain groups were impaired by PVC leachate exposure, others were stimulated and an overall increase in heterotrophic bacterial cell counts and relative abundance of specific bacteria, particularly Alteromonadales and Oceanospirillales, indicate that some bacteria may benefit from exposure to PVC leachates. Given the widespread impact of plastic pollution in marine habitats, fundamental changes in microbial community assemblage structure and function of this type have the potential to re-shape marine trophodynamics and biogeochemical cycling.

## Supplementary Information

**Additional file 1: Figure S1.** Microcosm experimental design. PVC plastic leachate was prepared by cutting PVC matting into small pieces, leaching them in Turks Island Salt solution for 5 days, and 0.2 μm filtering to remove plastic pieces and microorganisms from the PVC leachate solution. Experimental bottles containing seawater were set up by adding the PVC leachate in two different concentrations for 1% PVC leachate and 10% PVC leachate treatments, adding ZnCl2 in two concentrations for ZnL (0.13 mg L-1) and ZnH (1.3 mg L-1) treatments, and having one no addition seawater control. Quadruplicate replicate bottles were set up for each treatment and the control and incubated in an outside tank with light and temperature to mimic the conditions of the seawater source (issues with two ZnH treatment bottles meant they were not able to be included in the final analysis). **Figure S2.** Gating strategies used to quantify photosynthetic communities via flow cytometry analysis. A representative sample of a natural population (panel 1) illustrates how Chlorophyll and phycoerythrin (PE) autofluorescence were used to discriminate two groups of pigment-containing organisms: photosynthetic eukaryotes and Synechococcus. Specifically, eukaryotes were distinguished from Synechococcus based on their high Chlorophyll autofluorescence and relatively low PE fluorescence (panel 2). From there different eukaryotic communities were then discriminated based on FSC and PE content (panel 3). Synechococcus communities were discriminated based on PE and SSC content (panel 4). The gating strategy outlined was used for all of the samples. **Figure S3.** Gating strategies used to quantify heterotrophic bacterial and viral communities via flow cytometric analysis. A representative sample of a natural population (panel 1) illustrates how Sybr Green I fluorescence intensity and violet SSC were used to discriminate viral and bacterial communities. Contour plots of these two communities enabled identification of 2 different populations of bacteria, low DNA-containing bacteria (LDNAb) and high DNA-containing bacteria (HDNAb) (panel 2), and 5 distinct viral populations (panel 3). The gating strategy outlined was used for all the samples. **Figure S4.** Impact of PVC plastic leachate and zinc treatments on various flow cytometric populations. Changes in (a) photosynthetic Nano-eukaryote (NEUK) and phycoerythrin-rich eukaryote (PeEUK) population abundances and in (b) the ratio of Synechococcus to total eukaryotic primary producers community abundances are shown during the 6-day experiment. The effect of the different treatments on (c) the ratios between HDNAb vs LDNAb bacterial communities (HL_ratio) and total viral to total bacterial communities (VB_ratio), and their impact on (d) the five different viral populations are presented over the 6-day experiment. Data points are derived from the daily mean flow-cytometrically derived abundance (cells ml-1) of quadruplicate, biologically-independent samples (except ZnH for which just 2 replicates are available) for each day of the 6-day experiment. Error bars on each point represent the standard deviation. The lower-case letters (a-c) on panels a, b and c indicate which treatments were found to be statistically different (*p* < 0.01) from each other at the final time point (Additional file 2: Table S5). Significance was calculated with an ANOVA test followed by pairwise t-test and p-value false discovery rate (FDR) corrected. **Figure S5.** Composition of the bacterial and eukaryotic communities before (T0) and after exposure (day six) to PVC leachate and zinc. Boxplots indicate the average Shannon diversity for the (a) bacterial and (b) eukaryotic communities for each of the treatments. Different lower-case letters within the panels indicate a significance difference (*p* < 0.01, FDR corrected, Additional file 2: Table S7) from the control in the Shannon diversity. Non-metric multidimensional scaling (nMDS) plots are based on a Bray-Curtis dissimilarity matrix of the rarefied (c) bacterial and (d) eukaryotic community composition for each treatment bottle with axes indicating the percentage of explanation for the first two components. Stacked bar plots show the shift of (e) bacterial classes that contribute to > 1% of the total relative abundance per samples and the shift of (f) eukaryotic phyla that contribute to > 1% of the total relative abundance across treatments. The communities experienced changes in the relative abundance of some bacterial groups during the course of the experiment (from T0 to SW on day six in Fig. S5), likely due to bottle effects; however, these changes were less pronounced than the treatment effects. **Figure S6.** Bacterial community composition across the different treatments. Stacked bar plots show the bacterial Amplicon Sequence Variants (ASVs) that contribute to > 1% of the total relative abundance for seawater replicates at T0 and each treatment sample replicate at day 6. **Figure S7.** Eukaryotic community composition across the different treatments. Stacked bar plots show the eukaryotic Amplicon Sequence Variants (ASVs) that contribute to > 1% of the total relative abundance for seawater replicates at T0 and each treatment sample replicate at day 6. **Figure S8.** Average doubling times (h) for the isolated bacterial MAGs aggregated at the family level. Doubling times were calculated based on codon frequencies of multiple, highly expressed ribosomal genes for each MAG following the R package gRodon.

**Additional file 2: Table S1.** Flow cytometric counts (cells mL-1) and forward scatter (FSC) of Synechococcus and two photosynthetic eukaryotic populations along with the ratio of the Synechococcus to total photosynthetic eukaryote populations. **Table S2.** Flow cytometric counts (cells mL-1) of bacterial and viral subpopulations and total populations along with the ratio of total virus populations to total bacterial populations. **Table S3.** Statistics for changes in flow cytometrically-quantified population abundances (count) of various populations at experimental day 6 for different treatments. **Table S4.** Measurements of photosynthetic efficiency for each treatment during the 6 days of the experiment. **Table S5.** (a) Concentrations of all of the metal tested with ICP-MS. Metal concentration is espressed in mg/L. Highlighted in green are the metal for which the concentration was above the limit of the detection for at least one of the treatment or for the seawater control. ICP-MS analysis was performed at the Elemental Analysis Facility at the Southern Cross University in Lismore,NSW (Australia) an accredited NATA facility for the analysis of seawater samples. Methods reference has the code for the NATA standard methodology followed. T0 indicates samples that were collected at the beginning of the experiment, day 6 samples that were collected at the end of the experiment . *PQL= Minimum dectection limit. (b) Concentrations of all of the metal tested with ICP-MS for the preliminary batch of PVC. Metal concentration is espressed in ug/L. Highlighted in green are the metal for which the concentration was above the limit of the detection for at least one of the replicate. ICP-MS analysis was performed at the Elemental Analysis Facility at the Southern Cross University in Lismore,NSW (Australia) an accredited NATA facility for the analysis of seawater samples. **Table S6.** Statistical test results for community profile groupings based on amplicon analyses. **Table S7.** Alpha diversity calculations for the bacterial and eukaryotic communities based on amplicon analyses. **Table S8.** DESeq2 results for the bacterial community. Only results with an alpha < 0.01 were chosen. **Table S9.** Anova followed by pairwise t-test FDR corrected calculated for the photosynthetic groups identified based on cyanobacteria and plastid sequences from 16S rRNA. **Table S10.** DESeq2 results for the eukaryotic community. Only results with an alpha < 0.01 were chosen. **Table S11.** Taxonomic assignment of reads for genes in the bacterial metagenomes and the statistical analyses of the taxonomic groups in each treatment. **Table S12.** DESeq2 results for PVC10 vs SW on day 6. Only KEGG orthologs with log2Fold change >2 and < -2 are displayed. **Table S13.** DESeq2 results for PVC1 vs SW on day 6. Only KEGG orthologs with log2Fold change >2 and < -2 are displayed. **Table S14.** DESeq2 results for ZnH vs SW on day 6. Only KEGG orthologs with log2Fold change >2 and < -2 are displayed. **Table S15.** DESeq2 results for ZnL vs SW on day 6. Only KEGG orthologs with log2Fold change >2 and < -2 are displayed. **Table S16.** Pairwise comparison of the abundance of integrase genes in the different treatments. Only significant comparisons (p.adj < 0.05) are reported here. Significance was calculated with an ANOVA followed by a pairwise t-test and the p value FDR adjusted. n1 and n2 represents the number of replicates for treatment group 1 and group 2 respectively. **Table S17.** (a) Summary information on the assembled metagenome-assembled genomes (bins) and MAGs coverage in the different samples. (b) Pairwise test on the MAGs distribution between the different treatments, p-value was calculate in R with the r-statix package and FDR corrected. **Table S18.** Pairwise comparison of the coverage of almost complete metabolic pathway modules of the identified MAGs (completion >49%) across the different treatments. Significance was calculated with an ANOVA followed by a pairwise t-test and the pvalue FDR adjusted. n1 and n2 represents the number of replicates for group 1 and group 2 respectively.

## Data Availability

Data presented in this manuscript are available on the NCBI data portal under bioproject PRJNA756323. All data needed to evaluate the conclusion of the study (flow cytometry, photosynthetic efficiency, taxonomic and functional abundance tables) are available as supplementary materials.
