# Supplementary material for: Plastic leachates impair picophytoplankton and dramatically reshape the marine microbiome
Source: Microbiome. 2022 Oct 24;10:179. doi: 10.1186/s40168-022-01369-x (PMC9590215; doi:10.1186/s40168-022-01369-x)
Supplement: Supplementary file 2 — Additional file 1: Figure S1. Microcosm experimental design. PVC plastic leachate was prepared by cutting PVC matting into small pieces, leaching them in Turks Island Salt solution for 5 days, and 0.2 μm filtering to remove plastic pieces and microorganisms from the PVC leachate solution. Experimental bottles containing seawater were set up by adding the PVC leachate in two different concentrations for 1% PVC leachate and 10% PVC leachate treatments, adding ZnCl2 in two concentrations for ZnL (0.13 mg L-1) and ZnH (1.3 mg L-1) treatments, and having one no addition seawater control. Quadruplicate replicate bottles were set up for each treatment and the control and incubated in an outside tank with light and temperature to mimic the conditions of the seawater source (issues with two ZnH treatment bottles meant they were not able to be included in the final analysis). Figure S2. Gating strategies used to quantify photosynthetic communities via flow cytometry analysis. A representative sample of a natural population (panel 1) illustrates how Chlorophyll and phycoerythrin (PE) autofluorescence were used to discriminate two groups of pigment-containing organisms: photosynthetic eukaryotes and Synechococcus. Specifically, eukaryotes were distinguished from Synechococcus based on their high Chlorophyll autofluorescence and relatively low PE fluorescence (panel 2). From there different eukaryotic communities were then discriminated based on FSC and PE content (panel 3). Synechococcus communities were discriminated based on PE and SSC content (panel 4). The gating strategy outlined was used for all of the samples. Figure S3. Gating strategies used to quantify heterotrophic bacterial and viral communities via flow cytometric analysis. A representative sample of a natural population (panel 1) illustrates how Sybr Green I fluorescence intensity and violet SSC were used to discriminate viral and bacterial communities. Contour plots of these two communities enabled identification [file 40168_2022_1369_MOESM1_ESM.docx]

Supplementary Figures for:

**Plastic leachates impair picophytoplankton and dramatically reshape the marine microbiome**

**Authors:**

Amaranta Focardi^1*^, Lisa R. Moore^2^, Jean-Baptiste Raina^1^, Justin R. Seymour^1^, Ian T. Paulsen^2,3^, Sasha G. Tetu^2,3*^

**Affiliations:**

^1^ Climate change cluster (C3), University of technology Sydney, Sydney

^2^ Molecular Science department, Macquarie University, Sydney

^3^ ARC Centre of Excellence in Synthetic Biology

*Corresponding authors:

Amaranta Focardi^1^, email: [Amaranta.focardi@uts.edu.au](mailto:Amaranta.focardi@uts.edu.au)

Sasha G.Tetu^2^, email: sasha.tetu@mq.edu.au


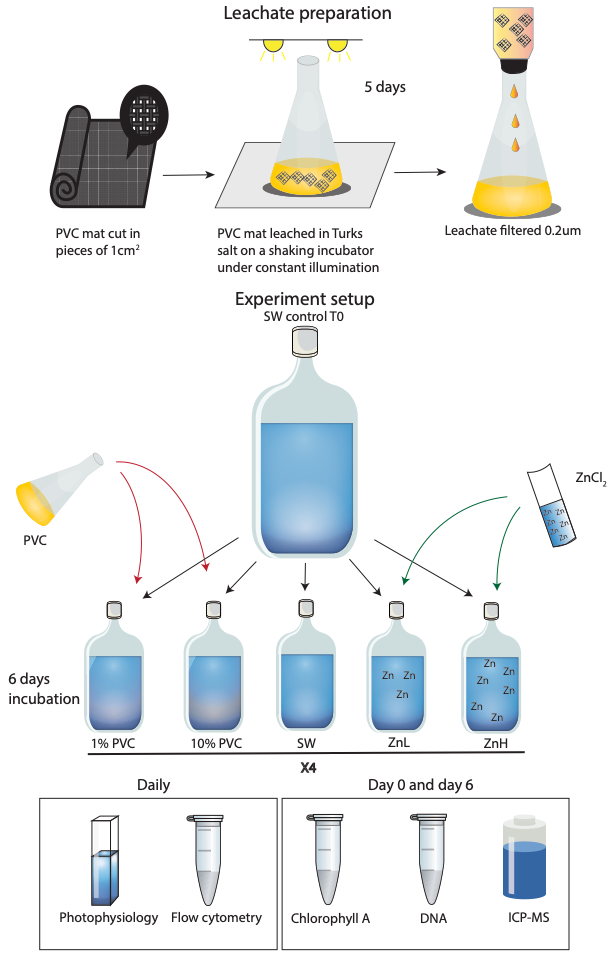


**Suppl. Figure 1** Microcosm experimental design. PVC plastic leachate was prepared by cutting PVC matting into small pieces, leaching them in Turks Island Salt solution for 5 days, and 0.2 μm filtering to remove plastic pieces and to sterilize the PVC leachate solution. Experimental bottles containing seawater were set up by adding the PVC leachate in two different concentrations for 1% PVC leachate and 10% PVC leachate treatments, adding ZnCl_2_ in two concentrations for ZnL (0.13 mg L^-1^) and ZnH (1.3 mg L^-1^) treatments, and having one no addition seawater control. Quadruplicate replicate bottles were set up for each treatment and the control and incubated in an outside tank with light and temperature to mimic the conditions of the seawater source (issues with two ZnH treatment bottles meant they were not able to be included in the final analysis).

**Suppl. Figure 2** Gating strategies used to quantify photosynthetic communities via flow cytometry analysis. A representative sample of a natural population (panel 1) illustrates how Chlorophyll and phycoerythrin (PE) autofluorescence were used to discriminate two groups of pigment-containing organisms: photosynthetic eukaryotes and *Synechococcus*. Specifically, eukaryotes were distinguished from *Synechococcus* based on their high Chlorophyll autofluorescence and relatively low PE fluorescence (panel 2). From there different eukaryotic communities were then discriminated based on FSC and PE content (panel 3). *Synechococcus* communities were discriminated based on PE and SSC content (panel 4). The gating strategy outlined was used for all of the samples.

**Suppl. Figure 3** Gating strategies used to quantify heterotrophic bacterial and viral communities via flow cytometric analysis. A representative sample of a natural population (panel 1) illustrates how Sybr Green I fluorescence intensity and violet SSC were used to discriminate viral and bacterial communities. Contour plots of these two communities enabled identification of 2 different populations of bacteria, low DNA-containing bacteria (LDNAb) and high DNA-containing bacteria (HDNAb) (panel 2), and 5 distinct viral populations (panel 3). The gating strategy outlined was used for all the samples.

**Suppl. Figure 4** Impact of PVC plastic leachate and zinc treatments on various flow cytometric populations. Changes in (a) photosynthetic Nano-eukaryote (*NEUK*) and phycoerythrin-rich eukaryote (PeEUK) population abundances and in (b) the ratio of *Synechococcus* to total eukaryotic primary producers community abundances are shown during the 6-day experiment. The effect of the different treatments on (c) the ratios between HDNAb vs LDNAb bacterial communities (HL_ratio) and total viral to total bacterial communities (VB_ratio), and their impact on (d) the five different viral populations are presented over the 6-day experiment. Data points are derived from the daily mean flow-cytometrically derived abundance (cells ml^-1^) of quadruplicate, biologically-independent samples (except ZnH for which just 2 replicates are available) for each day of the 6-day experiment. Error bars on each point represent the standard deviation. The lower-case letters (a-c) on panels a, b and c indicate which treatments were found to be statistically different (p < 0.01) from each other at the final time point (Suppl. Table 5). Significance was calculated with an ANOVA test followed by pairwise t-test and p-value false discovery rate (FDR) corrected.


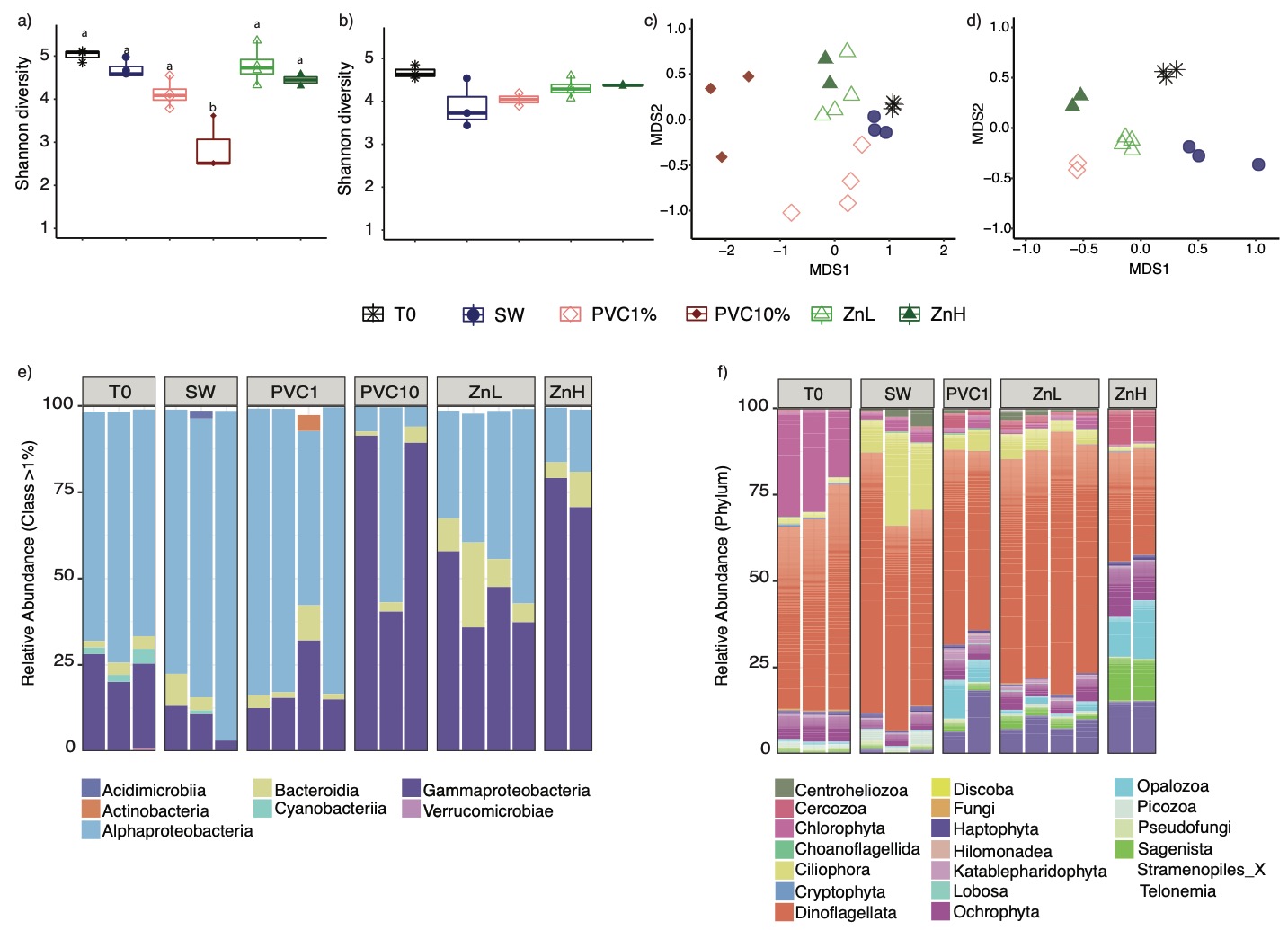


**Suppl. Figure 5** Composition of the bacterial and eukaryotic communities before (T0) and after exposure (day six) to PVC leachate and zinc. Boxplots indicate the average Shannon diversity for the (a) bacterial and (b) eukaryotic communities for each of the treatments. Different lower-case letters within the panels indicate a significance difference (p < 0.01, FDR corrected, Suppl. Table 7) from the control in the Shannon diversity. Non-metric multidimensional scaling (nMDS) plots are based on a Bray-Curtis dissimilarity matrix of the rarefied (c) bacterial and (d) eukaryotic community composition for each treatment bottle with axes indicating the percentage of explanation for the first two components. Stacked bar plots show the shift of (e) bacterial classes that contribute to > 1% of the total relative abundance per samples and the shift of (f) eukaryotic phyla that contribute to > 1% of the total relative abundance across treatments. The communities experienced changes in the relative abundance of some bacterial groups during the course of the experiment (from T0 to SW on day six in Suppl. Fig. 5), likely due to bottle effects^1,2^; however, these changes were less pronounced than the treatment effects.

**Suppl. Figure 6** Bacterial community composition across the different treatments**.** Stacked bar plots show the bacterial Amplicon Sequence Variants (ASVs) that contribute to > 1% of the total relative abundance for seawater replicates at T0 and each treatment sample replicate at day 6.

**Suppl. Figure 7** Eukaryotic community composition across the different treatments**.** Stacked bar plots show the eukaryotic Amplicon Sequence Variants (ASVs) that contribute to > 1% of the total relative abundance for seawater replicates at T0 and each treatment sample replicate at day 6.

**Suppl. Figure 8** Average doubling times (h) for the isolated bacterial MAGs aggregated at the family level. Doubling times were calculated based on codon frequencies of multiple, highly expressed ribosomal genes for each MAG following the R package gRodon.^3^

1. Agis, M., Granda, A. & Dolan, J. R. A cautionary note: Examples of possible microbial community dynamics in dilution grazing experiments. *J. Exp. Mar. Bio. Ecol.* **341**, 176–183 (2007).

2. Ferguson, R. L., Buckley, E. N. & Palumbo, A. V. Response of marine bacterioplankton to differential filtration and confinement. *Appl. Environ. Microbiol.* **47**, 49–55 (1984).

3. Weissman, J. L., Hou, S. & Fuhrman, J. A. Estimating maximal microbial growth rates from cultures , metagenomes , and single cells via codon usage patterns. **118**, 1–10 (2021).
